# Novel Neuroprotective Strategies in Ischemic Retinal Lesions

**DOI:** 10.3390/ijms11020544

**Published:** 2010-02-03

**Authors:** Krisztina Szabadfi, Laszlo Mester, Dora Reglodi, Peter Kiss, Norbert Babai, Boglarka Racz, Krisztina Kovacs, Aliz Szabo, Andrea Tamas, Robert Gabriel, Tamas Atlasz

**Affiliations:** 1 Department of Experimental Zoology and Neurobiology, University of Pecs, H-7624 Pecs, Hungary; E-Mails: kriszta.szabadfi@gmail.com (K.S.); mrtanki@cox.net (N.B.); gabriel@ttk.pte.hu (R.G.); 2 Department of Biochemistry and Medical Chemistry, University of Pecs, H-7624 Pecs, Hungary; E-Mails: laszlo.mester@aok.pte.hu (L.M.); boglarka.racz@aok.pte.hu (B.R.); krisztina.kovacs@aok.pte.hu (K.K.); aliz.szabo@aok.pte.hu (A.S.); 3 Department of Anatomy, University of Pecs, H-7624 Pecs, Hungary; E-Mails: dora.reglodi@aok.pte.hu (D.R.); peter.kiss@aok.pte.hu (P.K.); andrea.tamas@aok.pte.hu (A.T.); 4 Department of Sportbiology, University of Pecs, H-7624 Pecs, Hungary

**Keywords:** BCCAO, ischemia, retinoprotection, urocortin 2, diazoxide, PACAP, PARP-inhibitor, rat retina

## Abstract

Retinal ischemia can be effectively modeled by permanent bilateral common carotid artery occlusion, which leads to chronic hypoperfusion-induced degeneration in the entire rat retina. The complex pathways leading to retinal cell death offer a complex approach of neuroprotective strategies. In the present review we summarize recent findings with different neuroprotective candidate molecules. We describe the protective effects of intravitreal treatment with: (i) urocortin 2; (ii) a mitochondrial ATP-sensitive K^+^ channel opener, diazoxide; (iii) a neurotrophic factor, pituitary adenylate cyclase activating polypeptide; and (iv) a novel poly(ADP-ribose) polymerase inhibitor (HO3089). The retinoprotective effects are demonstrated with morphological description and effects on apoptotic pathways using molecular biological techniques.

## 1. Retinal Ischemia

The retina is supplied by two arterial systems: the chorocapillary layer supplies the outer retina, while the central retinal artery supplies the inner retina. The rich capillary networks provide an excellent blood supply suiting the high energy demand of the retinal light processing events. When the retinal circulation does not meet the requirements of the retina, the retina suffers an ischemic damage, present in numerous human conditions and leading to various degrees of visual impairment [1–6]. The pathways leading to ischemic retinal damage and the potential retinoprotective strategies have been reviewed in several excellent papers [1,7–10]. In the present review we focus on four recently proven retinoprotective agents in one type of animal model of retinal ischemia: in bilateral common carotid artery occlusion (BCCAO).

The main factors involved in ischemia-induced retinal degeneration are thought to be the excitatory neurotransmitter release, glial dysfunction, Ca^2+^ overload, formation of free radicals, elevation of nitric oxide and release of potentially toxic mediators by activated inflammatory cells such as tumor necrosis factor and interleukin-1 [1]. This complex cascade of events finally leads to degeneration of certain cell populations or the entire retina depending on the strength and duration of the ischemic event.

There are numerous animal models of retinal ischemia, including high intraocular pressure, ligation of the ophthalmic vessels and BCCAO with or without the occlusion of the vertebral arteries [1,11]. BCCAO leads to moderate reduction in the cerebral blood flow in rats leading to subtle changes in biochemical and behavioral measures. It has been shown that BCCAO causes long-lasting white matter lesion, neuronal degeneration, microglial activation, astrocytosis, behavioral deficits and changes in several biochemical parameters [12]. In the retina, the effects of chronic BCCAO depend on the rat strain and technique used. It produces varying degrees of retinal degeneration from subtle changes to severe degeneration, paralleling the retinopathy of carotid artery occlusive disease in humans [13,14]. Numerous electroretinographical, immunohistochemical, histological and functional studies show that BCCAO leads to varying degrees of ischemic damage of the retina [7,15,17–20].

**Figure 1.**
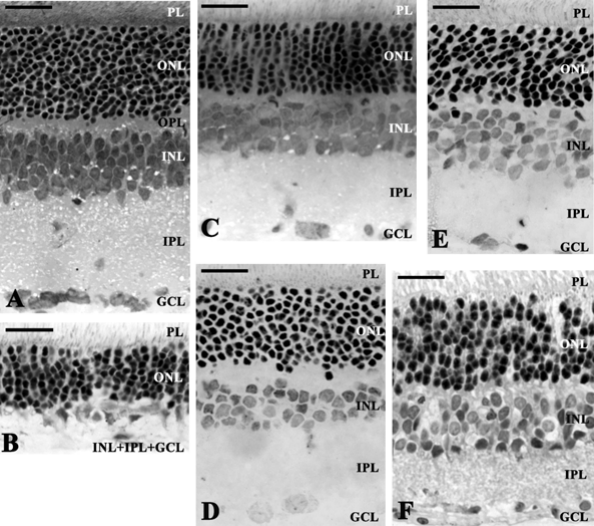
Microphotographs of different retinal sections. (A) Histological sections of control animals; (B) effects of bilateral common carotid artery occlusion (BCCAO). Extreme swelling of neuronal cell bodies and the fusion of the INL, IPL and GCL layers were observed. BCCAO caused severe overall retinal degeneration ameliorated by intravitreal injection of (C) urocortin 2, (D) diazoxide, (E) pituitary adenylate cyclase activating polypeptide and (F) the poly(ADP-ribose) polymerase inhibitor HO3089. Scale bar: 20 μm. Abbreviations: PL: photoreceptor layer; ONL: outer nuclear layer; OPL: outer plexiform layer; INL: inner nuclear layer; IPL: inner plexiform layer; GCL: ganglion cell layer.

**Figure 2.**
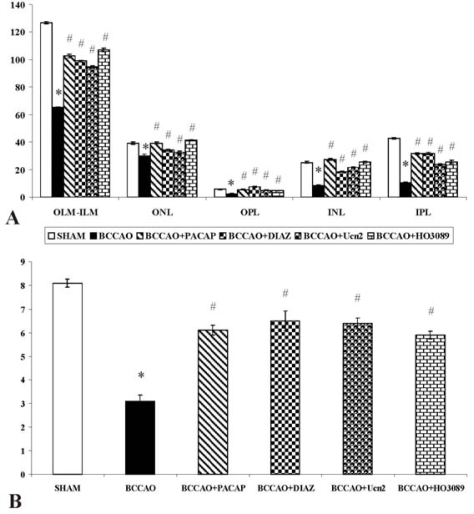
Quantification of the whole retina thickness (OLM-ILM), distinct retinal layers (A) and the cell number of GCL/100 μm in different conditions (B) by morphometrical analysis. Significant decreases were observed in BCCAO-induced retinal degeneration. The neuroprotective effects of urocortin 2; diazoxide; pituitary adenylate cyclase activating polypeptide and HO3089 treatments were quantified by the thickness of different retinal layers and also the cell number of GCL/100 μm. ^*^p < 0.05 between untreated and BCCAO; ^#^p < 0.05 BCCAO vs. BCCAO+different treatments. Results are presented as mean ± S.E.M. Statistical comparisons were made using the ANOVA test followed by Tukey-B`s post hoc analysis. Abbreviations: OLM: outer limiting membrane; ONL: outer nuclear layer; OPL: outer plexiform layer; INL: inner nuclear layer; IPL: inner plexiform layer; GCL: ganglion cell layer; ILM: inner limiting membrane.

Previously, we have found that permanent BCCAO leads to a severe retinal damage, with all retinal layers bearing the marks of deterioration [20,21]. The most marked reduction in thickness can be observed in the plexiform layers, and as a consequence, the distance between outer limiting membrane and inner limiting membrane is significantly less than in control preparations (Figures 1A, 1B and 2). The photoreceptor layer also suffers degeneration: the outer segments become shorter and the geometric arrangement is disturbed. Numerous photoreceptors and possibly the second- and third-order neurons belonging to the same retinal circuitry have been found to be damaged. This assumption is further justified by our observation of degenerating bipolar cell terminals in the inner plexiform layer.

## 2. Potential Protective Strategies

Given the complexity of events leading to retinal cell death, a variety of pharmacological approaches has been shown to be protective in retinal ischemia. As glutamate-mediated excitotoxicity is one of the main factors in retinal ischemia, decreasing excitotoxicity is an important therapeutic approach [1]. Other strategies involve reducing the detrimental effects of free radicals and increased Ca^2+^ levels, counteracting mitochondrial failure, anti-inflammatory strategies and potentiating endogenous protective mechanisms [1,22]. Table 1 gives a brief overview of the protective strategies proven in animal models of retinal ischemia. In the following sections, we describe four novel neuroprotective strategies recently found to reduce ischemic retinal damage in the rat.

## 3. Urocortin 2

Urocortins (Ucn 1, 2 and 3) are paralogs of corticotropin-releasing factor (CRF) [52]. In terms of primary structure, Ucn 2 most resembles Ucn 3, with both peptides acting as a preferential or selective CRF2 agonist, leading to their designation as the selective CRF2 agonists, Ucn 2 and Ucn 3 [53]. Ucns have been proposed to participate in many physiological functions, including anxiety, learning and memory, osmoregulation, thermoregulation, feeding, reproductive and cardiovascular functions [52,54–58]. Ucns confer protection when added to post-ischemic/hypoxic cardiomyocytes or to isolated intact heart during reperfusion after regional ischemia, preventing necrotic and apoptotic cell death and reducing infarct size, respectively [52,59–62]. Ucn 1 and 2 are known to exert cardioprotective effects against ischemic/hypoxic insults via a CRF2 dependent mechanism [52]. The neuroprotective activity of Ucn 1 is mediated by CRF1 receptors via cAMP-dependent pathways [63]. Less is known about the neuroprotective functions of Ucns. Ucn 1 has been shown to protect hippocampal neurons against excitotoxic and oxidative injuries [64]. mRNA transcripts for both CRF receptor subtypes, class B G-protein coupled receptors [65,66], and the CRF peptides also have been reported in retina [67–72], so further studies are needed to determine the mediating receptor subtype. The downstream mechanisms underlying Ucn 2 retinoprotection also remain uncertain. CRF-like immunoreactivity is present in amacrine, horizontal, and ganglion cells, as well as inner and outer nuclear and plexiform layers. The presence of CRF, Ucns, POMC and mRNAs of prohormone convertases 1 and 2 also has been shown in the retinal pigment epithelium [66]. Based on the retinal distribution of the CRF peptide family, it has been suggested that ocular tissues express CRF/Ucn-driven signaling systems that may play multiple roles in retinal function [66]. We have provided evidence that acute intravitreal Ucn 2 administration attenuates the marked degeneration of retinal layers that otherwise is seen two weeks following permanent BCCAO in rats. The *in vivo* findings demonstrate that protective actions of Ucn 2 extend to sparing retina from ischemic injury (Figures 1C and 2) [73].

## 4. Diazoxide

Mitochondrial dysfunction is involved in many key events of neuronal cell death in the retina [1]. ATP-sensitive K^+^ channels are located in different parts of the cell, including the inner mitochondrial membrane. 7-chloro-3-methyl-4H-1,2,4-benzothiadiazine-1,1-dioxide, diazoxide (DIAZ), a selective mitochondrial, ATP-dependent K^+^ channel opener that has been implicated in cytoprotection in cardiac and cerebral ischemia [12]. The cardio- and neuroprotective effects of various agents are attributed to the activation of these channels [74,75]. This type of neuroprotection represents a new mechanism of protection which is not dependent on blocking glutamatergic receptors or scavanging free radicals [74]. DIAZ is usually applied as pretreatment before CNS insults because the drug is known to mimic the effects of ischemic preconditioning [76–79]. When DIAZ is used prior to the insult *in vitro*, it protects against neuronal cell death induced by oxidative stress or glutamate [80,81]. DIAZ can target both mitochondrial and surface cation channels on the astrocytes, thereby modulating the astrocytic function [82]. DIAZ has mostly been applied in neuronal cell cultures exposed to oxygen–glucose deprivation [74,81,83–86]. *In vivo*, it has neuroprotective effects in various cerebral ischemic experimental conditions [74,83,87,88–90]. The protective effects of DIAZ have also been described both using pre- and postischemic administration in BCCAO [88,89,91,92], but relatively little is known about its putative protective effects in the retina. The mitochondrial ATP-sensitive K^+^ channels are also present in the retina, where the stimulatory effects of DIAZ have been reported [93]. It has been shown that DIAZ enhances survival of retinal ganglion cells, protects retinal neurons against excitotoxicity and inhibits the glutamate-induced mitochondrial depolarization *in vitro* [75,94]. DIAZ has also been reported to block the hypoxia-induced horizontal cell depolarization and the reduction of the light-evoked hyperpolarization *in vitro* [95]. *In vivo*, ischemic preconditioning can effectively be mimicked by DIAZ [96]. In an *in vitro* system, opening the mitochondrial K^+^ channels has been shown to inhibit the oxygen/glucose deprivation-induced glutamate release and to be protective in a model of retinal ischemia [97]. In a superfused retinal system, DIAZ has blocked the hypoxia-induced horizontal cell depolarization and the reduction of the light-evoked hyperpolarization [95]. We have recently reported that local administration of DIAZ is protective in retinal degeneration induced by neonatal monosodium-glutamate treatment or by BCCAO-induced ischemic damage of the retina (Figures 1D and 2) [98]. The mechanism could be multiple, including acute cytoprotective effects of the drug as well as early and late preconditioning. If DIAZ is available for the cells at the time of the ischemia/hypoxia or other kind of depolarization, its protective mechanism can be mediated by reduction of the mitochondrial calcium load [83].

## 5. Pituitary Adenylate Cyclase Activating Polypeptide

An important approach in neuroprotection is to potentiate or mimic endogenous protective mechanisms [1,96]. Several trophic factors have protective effects against retinal ischemia. Intravitreal injections of brain-derived neurotrophic factor, ciliary neurotrophic factor, basic fibroblast growth factor, hepatocyte growth factor and pigment epithelium derived factor result in significantly less damage in the inner retinal layers [99–101]. Pituitary adenylate cyclase activating polypeptide (PACAP) is a neurotrophic and neuroprotective peptide that has been shown to exert protective effects in different neuronal injuries, such as traumatic brain and spinal cord injury, models of neurodegenerative diseases and cerebral ischemia [102–104]. PACAP and its receptors are present in the retina [105,106]. PACAP is also a trophic factor in the nervous system and retina [107–109]. Increasing body of evidence shows that PACAP is an effective protective agent in retinal injuries. *In vitro*, the peptide has been shown to counteract the excitotoxic lesion induced by glutamate [110], cell death induced by anisomycin [111] and electrophysiological changes induced by anoxia [112]. *In vivo*, PACAP protects the retina against glutamate and kainate toxicity and optic nerve transection [113–118].

The neuroprotective effects of PACAP seem to be mediated predominantly by PAC1 receptors, involving protein kinase A and C (PKA and PKC) pathways. A major contribution to this effect has been shown to come from the PKA/MAPK (mitogen activated protein kinase) pathway and downstream, the inhibition of the apoptosis executor, caspase-3. In the retina, a part of this complex neuroprotective mechanism has been confirmed in an *in vivo* model, the monosodium-glutamate induced degeneration. PACAP has been shown to upregulate the antiapoptotic pathways, such as PKA, cAMP response element binding (CREB) and extracellular signal-regulated kinase (ERK) phosphorylation, and the PKA/Bad/14-3-3 protein cascade resulting in increased expression of the protective Bcl-xL and Bcl-2 [119–121]. At the same time, PACAP treatment downregulates the proapoptotic signaling, such c-Jun N-terminal kinase (JNK), apoptosis inducing factor (AIF), caspase-3, and the release of mitochondrial cytochrome c into the cytosol [119–121].

Recently, we have provided evidence that PACAP also reduces ischemic damage of the retina, protecting all inner retinal layers (Figures 1E and 2) [117,122]. This correlates with previous results showing the distribution of PAC1 receptor in the retina [105]. Strongest expression of the receptor is found in the GCL and INL, while weaker expressions are found in the ONL and OPL. This pattern of receptor expression provides basis for the sites of action by intraocular PACAP administration in our studies. Also, the PACAP antagonist PACAP6-38 could counteract the protective effects of PACAP mostly in the INL and GCL, where the strongest receptor expression has been described.

## 6. PARP Inhibition

The multifunctional nuclear enzyme, poly(ADP-ribose) polymerase (PARP) is implicated as a major regulator of the cell death process induced by a variety of environmental stimuli [123]. It is well established that overproduction of reactive oxygen species in response to the environmental stimuli cause DNA damage that leads to PARP activation [124]. Excessive PARP activation results in depletion of its substrate, NAD^+^ leading to ATP depletion and necrotic cell death as a consequence of energy loss. PARP activation facilitates other components of the cell death machinery too, namely; destabilization of the mitochondrial membrane systems [125,126], nuclear translocation of AIF [127] and activation of cell death promoting kinases such as JNK [128]. In addition, PARP activation suppresses the cytoprotective phosphatidyl-inositol-3 kinase-Akt pathway [129]. In the retina, increased activation of PARP contributes to retinal ganglion cell death in response to optic nerve transection [130], is involved in photoreceptor degeneration in the retinal degeneration-1 transgenic mouse model [131] and oxidative stress-induced apoptosis of ganglion cells [132]. PARP inhibition, on the other hand, has been demonstrated to decrease retinal damage in NMDA-induced cell death in the retina [133] and N-methyl-N-nitrosourea-induced photoreceptor cell apoptosis [134].

Although involvement of PARP activation in various ischemia models has been thoroughly studied [135–137], only circumstantial evidences are available for the role of PARP activation in chronic hypoperfusion induced neurodegenerative processes [138]. We have recently provided evidence that a novel PARP inhibitor HO3089 suppresses the morphological and biochemical changes induced by chronic hypoperfusion [20]. Upon PARP inhibitor treatment, the normal morphological structure of the retina was preserved and the thickness of the retinal layers was increased compared to the control ischemic eyes (Figures 1F and 2). Western blot analysis revealed activation of poly-ADP-ribose (PAR) synthesis, which was inhibited by the PARP inhibitor indicating that PARP activation was a causative factor behind the hypoperfusion-induced retinal degeneration. Inhibition of PARP also led to increased activation of one of the most important cytoprotective pathways, the phosphatidyl-inositol-3 kinase-Akt system and its downstream target, GSK-3beta. The signal transduction pathways involving MAP kinases play key roles in cellular survival and adaptation in the retina. BCCAO induced phosphorylation of JNK and p38 MAPK. HO3089 decreased the phosphorylation of these proapoptotic MAPKs. In addition, HO3089 treatment induced phosphorylation that is activation, of the protective ERK signaling pathways [20].

## 7. Conclusions

In the present review we have summarized recent findings describing novel neuroprotective strategies in ischemic retinal lesions induced by chronic BCCAO. The described strategies encompass four substances acting on different protective pathways, such as endogenous neurotrophism (PACAP and Ucn 2), mitochondrial integrity (DIAZ) and PARP inhibition. The vast amount of retinoprotective agents proven to be effective in animal models provides a divergent array of possible therapeutic strategies in ischemic retinal injury. Further studies are necessary to determine the most effective combination of putative therapeutic treatments in human retinal diseases. The perspective we illustrate by histological studies may also yield novel perspectives on other hypoxic-ischemic retinal disorders, including diabetic retinopathy and age-related macular degeneration, diseases, which are characterized by both vascular and neural abnormalities of the retina.

## Figures and Tables

**Table 1.** Brief overview of potential retinoprotective strategies proven in animal models of retinal ischemia.

***Substance***	***Main effects***	***References***
Antioxidants (e.g., Vitamin E, lutein, flavonoids)	↓ oxidative damage ↓ caspase3 ↑ glutathione ↓ nitrotyrosine ↓ nuclear PAR ↓ loss of ATP	[23] [24] [25]
Ischemic preconditioning	protein kinase C activation ATP-sensitive K^+^ channel opening ↑ ferritin level adenosine A1 receptor stimulation STAT-3 activation ↑ HSP27	[26] [27] [28] [29] [30]
Ischemic postconditioning	↓ glutamate	[31]
Adenosine	vasodilation ↓ neuronal activity ↑ glycogenolysis	[32]
Growth factors (IGFII, NGF, BDNF, VEGF)	↑ phosphate activated glutaminase (PAG) ↓ ammonia ↑ blood flow to the retina ↑ Bcl-2 ↓ Bax	[33] [34] [35]
Erythropoietin	↓ apoptosis ↑ ischemic preconditioning	[36] [37] [38]
Statins	↓ HSP27	[39]
Estradiol	↓ glutamate	[40]
Cannabinoids	↓ peroxynitrite	[41]
Morphine	↑ ischemic preconditioning	[42,43]
L-carnitine	↓ oxidative stress	[44]
Glutamate receptor antagonists	↓ glutamate excitotoxicity	[45]
Adrenergic receptor blockers	↓ influx of sodium and calcium	[46]
Alpha2 adrenergic agonist (brimonidine)	↓ glutamate and aspartate	[47]
Ca^2+^, K^+^, Na^+^ channel blockers	↓ influx of sodium and calcium ↑ ischemic preconditioning ↓ c-jun, p-JNK	[48,49]
Hypothermia	↓ energy demand	[50]
Hyperglycaemia	↑ HSP-27	[51]
